# Effectiveness of dry needling and injections of myofascial trigger points associated with plantar heel pain: a systematic review

**DOI:** 10.1186/1757-1146-3-18

**Published:** 2010-09-01

**Authors:** Matthew P Cotchett, Karl B Landorf, Shannon E Munteanu

**Affiliations:** 1 Department of Podiatry, Faculty of Health Sciences, La Trobe University, Bundoora, 3086, Australia; 2 Musculoskeletal Research Centre, Faculty of Health Sciences, La Trobe University, Bundoora, 3086, Australia

## Abstract

**Background:**

Plantar heel pain (plantar fasciitis) is one of the most common musculoskeletal pathologies of the foot. Plantar heel pain can be managed with dry needling and/or injection of myofascial trigger points (MTrPs) however the evidence for its effectiveness is uncertain. Therefore, we aimed to systematically review the current evidence for the effectiveness of dry needling and/or injections of MTrPs associated with plantar heel pain.

**Methods:**

We searched specific electronic databases (MEDLINE, EMBASE, AMED, CINAHL, SPORTDiscus and AMI) in April 2010 to identify randomised and non-randomised trials. We included trials where participants diagnosed with plantar heel pain were treated with dry needling and/or injections (local anaesthetics, steroids, Botulinum toxin A and saline) alone or in combination with acupuncture. Outcome measures that focussed on pain and function were extracted from the data. Trials were assessed for quality using the Quality Index tool.

**Results:**

Three quasi-experimental trials matched the inclusion criteria*: *two trials found a reduction in pain for the use of trigger point dry needling when combined with acupuncture and the third found a reduction in pain using 1% lidocaine injections when combined with physical therapy. However, the methodological quality of the three trials was poor, with Quality Index scores ranging form 7 to 12 out of a possible score of 27. A meta-analysis was not conducted because substantial heterogeneity was present between trials.

**Conclusions:**

There is limited evidence for the effectiveness of dry needling and/or injections of MTrPs associated with plantar heel pain. However, the poor quality and heterogeneous nature of the included studies precludes definitive conclusions being made. Importantly, this review highlights the need for future trials to use rigorous randomised controlled methodology with measures such as blinding to reduce bias. We also recommend that such trials adhere to the Standards for Reporting Interventions in Controlled Trials of Acupuncture (STRICTA) to ensure transparency.

## Background

Plantar heel pain (plantar fasciitis) is one of the most common musculoskeletal pathologies of the foot. It is estimated to affect 10% of the population at some time in their life [1], although there are few high quality epidemiological studies available. One national study of medical doctors in the United States during the years 1995 to 2000 found that approximately one million patient visits to physicians per year were for plantar heel pain [2]. In addition, a recent Australian study of 3206 adults found that approximately 20.9% (95% CI 17.7 to 24.45) indicated that they had heel pain, although this study did not differentiate between plantar heel pain and pain in other parts of the heel [3].

Plantar heel pain is generally accepted to predominantly affect middle aged as well as older adults. In a study of 784 North American community-dwelling residents aged 65 years or greater, 7% reported pain and tenderness beneath the heel [4]. Although plantar heel pain affects older adults, some other groups are also vulnerable. For example, it is also common in the athletic population, being estimated to contribute to 25% of all foot injuries related to running [5]. Plantar heel pain has been shown to have an impact on health-related quality of life. Individuals with chronic plantar heel pain experience social isolation; have a poor perception of their health status; are severely limited in their ability to undertake physical activities and lack the energy to undertake daily tasks [6].

Numerous interventions are used to treat plantar heel pain including calf stretching, foot taping, manual therapy (joint mobilisation and manipulation; mobilisation of soft tissue near sites of nerve entrapment and passive neural mobilisation techniques) foot orthoses, oral and injectable anti-inflammatories and night splints [7]. Surgery is recommended as a last resort and usually only after failure of at least six months of conservative therapy [8]. Clearly there are many interventions used to treat plantar heel pain, but the Clinical Practice Guidelines for plantar heel pain proposed by the Orthopaedic Section of the American Physical Therapy Association do not recommend one treatment over another [7]. Furthermore, two systematic reviews [9,10] have found few interventions that are supported by good evidence.

An alternative treatment for plantar heel pain involves dry needling and/or injections (local anaesthetics, steroids, Botulinum toxin A and/or saline) of myofascial trigger points (MTrPs) within the lower limb and foot. However, the aforementioned systematic reviews [9,10] did not identify any clinical trials that have investigated the effectiveness of dry needling and/or injections of MTrPs. Therefore, we aimed to systematically review the literature evaluating the effectiveness of dry needling and/or injections of MTrPs associated with plantar heel pain.

## Methods

### Types of studies

All clinical trials included in this review were obtained from peer-reviewed journals investigating the effectiveness of dry needling and/or injections of MTrPs associated with plantar heel pain. Randomised controlled and quasi-experimental (an experiment that lacks either randomisation of participants or control group(s) or both) trials examining the effectiveness of trigger point dry needling and/or injections for plantar heel pain were included. The decision to include quasi-experimental trials was based on the lack of randomised controlled trials to draw evidence from; hence we attempted to obtain an overview of what was known to date. Including non-randomised trials in systematic reviews can be appropriate when there are a limited number of randomised trials available [11]. Further, Linde et al.[12] conducted a systematic review of randomised and non-randomised trials that evaluated the effectiveness of acupuncture for chronic headache and found that non-randomised trials of good quality yielded positive responses to treatment that were similar to randomised-controlled trials. The authors concluded the inclusion of high quality non-randomised controlled trials into a systematic review might add to the generalisability of the findings. Letters to the editor, opinion pieces and editorials were excluded.

### Types of participants

A clinical trial was included if the participants were diagnosed with plantar heel pain. All participants were over the age of 18 and had experienced symptoms of any duration. A trial was only included if the participant's plantar heel pain was managed by treatment of MTrPs in the lower extremity and/or foot. The rationale for this decision was based on the assumption that some forms of plantar heel pain might occur secondary to MTrPs in plantar heel muscles (i.e. abductor hallucis and quadratus plantae) and/or referred pain from the soleus muscle [13]. A trial was excluded if the participant's plantar heel pain was associated with a vascular or neurological disease, arthritis (degenerative and inflammatory) or fibromyalgia.

### Types of Intervention

Clinical trials were included if they investigated the effectiveness of dry needling and/or injections (local anaesthetics, steroids, Botulinum toxin A and/or saline) of MTrPs for plantar heel pain. Trials were excluded if they involved needling of traditional acupuncture points as the sole treatment because the relationship between traditional acupuncture points and MTrPs is unclear [14]. However, it has been suggested that there might be a correlation between MTrPs and a class of acupuncture points referred to as Ah Shi points (pain points). Ah Shi points are a class of acupuncture points positioned outside the traditional Chinese meridians and are commonly treated by traditional acupuncturists for painful conditions including muscle spasm [15]. Given the uncertainty of this relationship, we included trials that utilised acupuncture only if it was combined with dry needling or injection of MTrPs.

### Types of outcome measures

A trial was included if any of the following primary outcome measures were used: Visual Analogue Scale; The Foot Health Status Questionnaire; The Foot Function Index or any other health-related quality of life measure. Secondary outcome measures investigating physiological changes (e.g. joint range of motion and pressure pain threshold) following the intervention were included providing at least one of the aforementioned primary outcome measures was reported.

### Search methods for identification of studies

In April 2010 the following electronic databases were used to search the literature: Ovid MEDLINE (1950 to date), Ovid EMBASE (from 1988 to date), Ovid AMED (from inception), CINAHL (1982 to date), SPORTDiscus (from inception) and AMI (1968 to date). A full electronic search strategy from the EMBASE database is included in Table 1.

**Table 1:** A full electronic search strategy from the EMBASE database, April, 2010

#	Searches
1	exp Lower Extremity/
2	exp Therapeutics/
3	exp Myofascial Pain Syndromes/
4	exp"Outcome and Process Assessment (Health Care)"/or exp"Quality of Life"/or exp"Outcome Assessment (Health Care)"/or exp Questionnaires/or exp Treatment Outcome
5	exp Heel Pain/or exp Pain Assessment/or exp Foot Pain/or exp Musculoskeletal Pain/
6	exp fasciitis/
7	exp methodology/
8	(leg* or calf or calves or foot or feet or ankle* or toe* or plantar fascia or plantar aponeurosis or plantar ligament or area).mp.
9	(needl* or acupuncture or inject*)
10	(trigger area* or trigger point* or"myofascial trigger point pain" or"myofascial pain components" or taut band).
11	(systematic review or"randomised controlled trial" or RCT or qausi experimental or"single subject design" or comparative study)
12	VAS or"visual analogue scale" or"visual analysis scale" or"activities of daily living" or"quality of life" or"pressure pain threshold" or algometry
13	9 or 2
14	6 or 3 or 10
15	5 or 12 or 4
16	11 or 7
17	1 or 8
18	13 and 14 and 15 and 16 and 17

In addition, experts in the field of MTrP therapy were questioned about their knowledge of further articles not captured in the database search. The reference lists of all included articles were hand searched for trials meeting the inclusion criteria. Finally, Google Scholar and SUMsearch were searched for grey literature (information that has not been published, or if published is not readily accessible). No language restrictions were applied.

### Study selection

Two investigators (MC and an impartial assessor) independently scanned the title and abstracts for information fulfilling the inclusion criteria. If a decision could not be made it was retained until the full text was obtained. A full text of all potentially eligible articles was then accessed and reviewed by both assessors to ensure eligibility. Discrepancies between the two reviewers were resolved using a third assessor (KBL).

### Data Extraction

A data extraction form (see Additional File 1) was modified from an existing standardised extraction form produced by the Centre for Reviews and Dissemination [16]. The content of the form included topics relevant to acupuncture and trigger point dry needling research as recommended by the Standards for Reporting Interventions in Controlled Trials of Acupuncture (STRICTA) [17]. Relevant data (means, mean differences, standard deviations, and p values) were extracted from the selected articles by two of the investigators (MC and SEM). Any disagreement between the authors was discussed with KBL and a general consensus agreed upon.

### Assessment of methodological quality

Two reviewers (MC and an impartial assessor) independently assessed the methodological quality of the included articles using the Quality Index (QI) [18] tool, which has been shown to have high internal consistency (KR-20: 0.89), good test-retest reliability (r = 0.88) and inter-rater reliability (r = 0.75). The original Quality Index is a 27-point checklist which covers four domains: internal validity, external validity, reporting and power. The literature has not established cut off values for the Quality Index methodological quality assessment tool. Downs and Black [18] (p. 381) stated that"the value of a single global score needs to be tested by reviewers making such an assessment before rather than after using the 27 item checklist". The use of a single summary score or global score has been criticised in the literature as it might eliminate sources of heterogeneity among the results [19].

For this systematic review, three items were modified. First, for Item 10, two points were allocated to trials that utilised confidence intervals as well as *p *values for the main outcomes as confidence intervals provide more information regarding the magnitude and precision of a treatment effect [20]. Second, Item 25 was removed as it has been shown that case mix adjustment cannot reduce the extent of bias in non-randomised trials [19]. Finally, Item 27 was removed as a minimally important difference using the visual analogue scale has not been calculated for MTrP interventions in participants with plantar heel pain.

## Results

**Figure 1:**
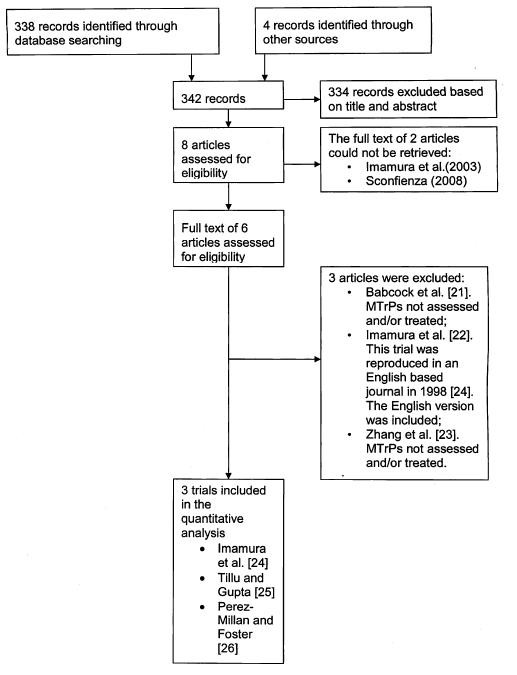
**Flow of information through the systematic review**.

A total of 342 studies were identified through database and other sources. Following inspection of the titles and abstracts, 334 were excluded. Of the 8 remaining studies, a full text of unpublished data (identified from conference abstracts) by Imamura et al. (2003) and Sconfienza (2008) could not be obtained from the authors. Further analysis of the full text from the remaining 6 studies resulted in 3 clinical trials fulfilling the inclusion criteria (Table 2) and 3 trials were excluded [21-23]. A flow diagram of the study selection process is presented in Figure 1.

**Table 2:** Characteristics of included studies

Trial	Design	Number allocated to experimental and control groups	Mean age in years (SD)	% Female	Mean duration of disease in months (SD)	Exclusion criteria	Criteria used to identify the MTrP
**Tillu and** **Gupta** **(1998)**	Quasi-experimental (one group)	Experimental = 18	49.1 (10.7)	72.3%	25.1 (10.7)	History of heel surgery or cortisone injection in last three months	No criteria used
							
**Imamura** **et al.** **(1998)**	Quasi-experimental (two groups, non-randomised)	Experimental = 15 (Actual number is unclear but it would appear that 20 were recruited and 5 dropped out) Control = 9 at discharge.	Experimental: 50.0 (12.2) Control: 44.0 (NR)	89.7%	27.0 (NR)	NR	MTrP identified via palpation (local tenderness and taut band)
							
**Perez-Millan** **and Foster** **(2001)**	Quasi-experimental (one group)	Experimental = 11	39.5 (12.7)	72.8%	39.0 (5.0)	NR	NR

Key: NR=Not reported

### Quality of the evidence

The inter-rater reliability of total Quality Index scores was not calculated due to the small number of trials included. Perfect agreement was recorded on all items except question 4 where there was 67% agreement between the assessors.

Table 3 presents the results from the quality assessment. All included studies were of a poor methodological quality. The total score of the Quality Index ranged from 7/27 to 12/27 with a mean Quality Index score of 10/27 across the three trials. The internal validity domain rated most poorly across the trials due to the presence of selection [24-26], detection [24], statistical [24], performance [24-26] and attrition bias [24]. In addition, all three trials used secondary outcome measures that were not valid and reliable.

**Table 3:** Evaluation of trial quality

Quality Index items	Imamura et al. (1998)	Tillu and Gupta (1998)	Perez Millan and Foster (2001)
**Reporting**			

1. Study hypothesis/aim/objective	1	1	1
2. Main outcomes	1	1	1
3. Characteristics of the participants	0	0	0
4. Interventions of interest	0	1	0
5. Distributions of principal confounders in each group	0	0	0
6. Main findings	0	1	1
7. Estimates of random variability for main outcomes	1	0	1
8. All the important adverse events that may be a consequence of intervention	0	0	0
9. Characteristics of patients lost to follow-up	0	1	1
10. Actual probability values for main outcomes	0	1	0

**External validity**			

11. Were subjects who were asked to participate representative of the entire population from which they were recruited?	1	1	0
12. Were subjects who were prepared to participate representative of the entire population from which they were recruited?	0	0	0
13. Were the staff, places, and facilities representative of the treatment the majority of subjects received?	1	0	1

**Internal validity (bias)**			

14. Was an attempt made to blind subjects to the intervention they received?	0	0	0
15. Was an attempt made to blind those measuring main outcomes of the intervention?	0	0	0
16. If any of the results of the study were based on"data dredging", was this made clear?	0	1	1
17. Do analyses adjust for different lengths of follow-up?	0	1	1
18. Were appropriate statistical tests used to assess the main outcomes?	1	1	1
19. Was compliance with the intervention reliable?	1	1	1
20. Were main outcome measures reliable and valid?	0	0	0

**Internal validity (selection bias)**			

21. Were patients in different intervention groups recruited from the same population?	0	0	0
22. Were subjects in different intervention groups recruited over the same period of time?	0	0	0
23. Were subjects randomized to intervention groups?	0	0	0
24. Was the randomized intervention assignment concealed from both patients and staff until recruitment was complete and irrevocable?	0	0	0
* 25. Was there adequate adjustment for confounding in the analyses from which main findings were drawn?	x	x	x
26. Were losses of subjects to follow-up taken into account?	0	1	1

**Power**			

* 27. Did the study have sufficient power to detect a clinically important effect where the probability for a difference due to chance was less than 5%?	x	x	x

**Total score (/27)**	**7**	**12**	**11**

Instructions for use

For Q 1-9 one point is allocated for Yes and zero points for No.

For Q 5 two points are allocated for Yes, one point for Partially and zero points for No.

For Q 10 two points are allocated for Yes, one point for Partially and zero points for No.

For Q 11-27 one point is allocated for Yes, zero points for No and zero points for Unable to Determine.

* Item removed.

### Trial characteristics

All trials had a quasi-experimental design with pre-test and post-test measures. Imamura et al.[24] conducted a quasi-experimental trial with a non-randomised control group to evaluate the effectiveness of 1% lidocaine injections of MTrPs in combination with physical therapy or conventional therapy alone within the foot and leg (Table 4). The physical therapy component included heat application for 20 minutes and faradic stimulation over the area treated for another 20 minutes. Stretching exercises were prescribed (3 times per day for 15 seconds) after heat application. In addition, relaxation exercises were issued to some participants if required. In contrast, the control group received conventional therapy, although the details were not included.

**Table 4:** Types of interventions, treatment regime and outcome measures

Trial	Intervention	Trigger points and Acupuncture points selected for treatment	Outcome measure	Number of treatment sessions per week
**Tillu and** **Gupta** **(1998)**	25 mm acupuncture needle (diameter unknown) inserted for 15 minutes and stimulated every 5 minutes for 5 sec. Needle was manipulated to produce *de qi*. No control group.	(i) Acupuncture points KI.3; BL.60 and SP.6 (ii) Gastrocnemius MTrP and heel MTrP. Specific location of MTrP in the heel and calf not identified.	(i) Visual analogue scale (ii) Verbal pain score Outcome measures recorded at 4 and 6 weeks post baseline.	4 sessions of acupuncture/1 per week. If symptoms were not resolved after this period, 2 sessions (1 per week) of acupuncture and dry needling were implemented.
				
**Imamura et al.** **(1998)**	22-25 gauge needle repetitively inserted and withdrawn with injection of 1% lidocaine into the MTrP; plus *standard therapy. Control group received conventional conservative therapy but not outlined in the methods.	Medial head of Gastrocnemius; Soleus; Tibialis posterior; Popliteus; Abductor hallucis; Peroneus Longus and Flexor digitorum brevis	(i) Duration of treatment (ii) Visual analogue scale (iii) Pressure pain threshold Outcome measures recorded at discharge, 6 and 24 months	The number of sessions and times per week varied between the groups
				
**Perez-Millan** **and Foster** **(2001)**	10-120 mm acupuncture needle (0.20-0.25 mm diameter); plus electrostimulator (2-4 Hz) for 20-30 minutes. No control group.	(i) Acupuncture points KI.1, 3, 6; BL.60, 67; GB 44 (ii) MTrPs points in the heel and arch regions	(i) Visual analogue scale (ii) **Foot function index questionnaire Outcome measures recorded at 6 weeks post baseline	6 sessions/1 per week

Key:

MTrP = myofascial trigger point.

*Standard therapy = implemented once per day for three days following the injection: included: (i) heat (20min) and faradic stimulation over affected area for 20 min, (ii) stretching for three days, 3 times per day for 15 seconds after hot pack application, (iii) participants advised to avoid walking and standing for two days post injection.

** Foot function index questionnaire used in this trial was not validated.

Tillu and Gupta investigated the effectiveness of a four-week course of traditional acupuncture followed by a two-week course of trigger point dry needling combined with acupuncture. This trial was not a cross-over design in the strict sense, rather all participants received the course of treatment in the same order. In contrast, Perez-Millan and Foster [26] investigated the effectiveness of trigger point needling combined with electro-acupuncture. Tillu and Gupta [25] and Perez-Millan and Foster [26] did not include a control group for comparison (refer to Table 4 for a description of the trigger point dry needling and injection details).

The characteristics used to identify a MTrP were not described by Tillu and Gupta [25] or Perez-Millan and Foster [26], however Imamura et al. [24] used the common criteria of a taut band and local tenderness to diagnose a MTrP. In addition, three trials varied in; the muscles that were treated; the size and type of needles used; the response elicited, and the duration of needle insertion. The treatment schedules were generally similar across the trials with weekly treatments for a period of six weeks. All three trials used a visual analogue scale as the primary outcome measure, although there was variability in the secondary outcome measures used.

### Evidence for the effectiveness of dry needling and/or injections of MTrPs associated with plantar heel pain

As clinical heterogeneity of the included trials was evident the findings of the included studies were combined using a narrative rather than a quantitative approach. As such, meta-analysis was not performed. Table 5 provides a detailed description of the mean differences between and within groups for the trial by Imamura et al. [24] and mean differences within groups for Tillu and Gupta [25] and Perez-Millan and Foster [26].

**Table 5:** Mean differences between and within groups of included studies

Trial	Difference between groups	Differences within groups		
**Tillu and** **Gupta** **(1998)**	N/A (one group only)	(i) VAS pain: @ 4 weeks (34.7% improvement, p < 0.001) @ 6 weeks (67.9% improvement, p < 0.001) @ 6 weeks vs 4 weeks, (difference 33.2%, p = 0.047)		
		(ii) Verbal pain score (% of improvement): 40.2 (40.1%) @ 4 weeks and 65.9 (32.8%) @ 6 weeks		
			**Intervention**	**Control**
**Imamura et** **al. (1998)**	Duration of treatment (weeks): Significantly less in intervention group (83.9% difference between the groups, p < 0.05)	(i) Mean duration of treatment in weeks (SD) (ii) VAS pain: @ discharge @ 6 months @ 2 years	3.4 (2.2) 58.4% improvement, p = 0.003 67.1% improvement, p = 0.007 67.1% improvement p = 0.002	21.1 (19.5) 54.9% improvement, p < 0.05 values not reported at 6 months values not reported at 12 months
		(iii) PPT (gastrocnemius): @ discharge @ 6 months @ 2 years	130% increase, p = 0.001 71% increase, p = 0.009 55% increase, p = 0.023	PPT not reported for control
		(iv) PPT (medial calcaneal tubercle) at: @ discharge @ 6 months @ 2 years	106% increase, p = 0.004 values not reported at 6 months 143% increase, p = 0.007	PPT not reported for control
**Perez-Millan** **and Foster** **(2001)**	N/A (one group only)	(i) VAS pain: @ 6 weeks (46% improvement, p < 0.001)		
		(ii) Foot function index questionnaire scores*: significantly less pain for 10 out of 12		

**Key**: N/A: Not applicable; VAS = visual analogue scale; MTrP = Myofascial trigger point; PPT = Pressure pain threshold; *The foot function index questionnaire used in this trial was not validated

Imamura et al. [24] found a statistically significant decrease in pain for the use of 1% lidocaine injections and standard therapy for the MTrP injection group at discharge (58.4% improvement, p = 0.003), six months (67.1% improvement, p = 0.007) and two years (67.1% improvement, p = 0.002). Similarly, a statistically significant decrease in pain was found for the control group at discharge (54.9% improvement, p < 0.05, the exact p value was not reported); however there was no follow-up at six months or two years for this group. Imamura et al. [24] found a statistically significant decrease in the duration of treatment between the injection and control groups (3.4 weeks versus 21.1 weeks respectively). Importantly the only between-group comparison made in this trial was for the total duration of treatment.

The other two trials by Tillu and Gupta [25] and Perez-Millan and Foster [26] only included a treatment group and no comparison was made to a control group. Nevertheless, Tillu and Gupta [25] observed a statistically significant improvement in pain for a two-week course of dry needling and acupuncture when compared to a previous four-week period of acupuncture treatment (p = 0.047). Finally, Perez-Millan and Foster [26] found a significant improvement in pain for the use of dry needling and electro-acupuncture (p < 0.001).

## Discussion

The aim of this study was to conduct a systematic review of the literature to evaluate the evidence for the effectiveness of dry needling and/or injections of MTrPs associated with plantar heel pain. The search strategy found three quasi-experimental trials. One trial compared the effectiveness of 1% lidocaine injections combined with standard therapy to standard therapy alone. A second trial evaluated the effectiveness of trigger point dry needling combined with electro-acupuncture, whereas a third trial evaluated the effectiveness of acupuncture followed by a period of acupuncture combined with trigger point dry needling. However, it is important to note that all trials were of poor methodological quality.

There were two major reasons for the low quality of the included trials. First, the internal validity of all three trials was potentially threatened. Tillu and Gupta [25] and Perez-Millan and Foster [26] did not include a control to compare the intervention to and therefore, the relationship between the dependent and independent variable might have been influenced by non-intervention effects, such as the natural course of the disorder. Imamura et al. [24] did compare the intervention to a control, however there was no evidence that the participants were randomised. Consequently, the two groups might not have been equivalent at baseline making it difficult to determine if the outcomes were a reflection of the intervention or differences in prognostic characteristics of the two groups at baseline. The internal validity of the trial by Imamura et al. [24] might have also been threatened due to a 25% loss of participants at discharge. As there was no reference to an intention-to-treat analysis the characteristics of the two groups may have become different as the trial progressed, which could have affected the estimate of the treatment effect. Further threats to internal validity might have occurred in all three trials, as no attempt was made to blind those responsible for measuring the outcomes.

Second, reporting of the trial rationale [24-26], eligibility criteria [25,26], study population [24-26], details of the researcher's background [24-26], needling and injection details [24-26], control intervention [24], and results [24-26], were all incomplete. Imamura et al. [24] did provide details of the muscles that were injected, however there was insufficient information which muscles were treated during each session, the number of injections (total and per muscle), and the depth of needle insertion. In addition, Tillu and Gupta [25] and Perez-Millan and Foster [26] did not report which muscles were dry needled in the foot, the number of needles inserted into a MTrP, the depth of needle insertion, or the needle response elicited during dry needling of a MTrP. The presence of a local twitch response during trigger point dry needling is suggested to help confirm the presence of a MTrP and is associated with a positive therapeutic outcome [27]. Furthermore, sensations described by the patient as a result of needling might be predictive of the analgesic response [28].

The reporting in two trials also failed to provide sufficient detail of the criteria used to identify a MTrP. While Imamura et al. [24] used the common criteria of a taut band and local tenderness to diagnose a MTrP, Tillu and Gupta [25] and Perez-Millan and Foster [26] did not provide any information regarding the diagnosis of a MTrP. As there is considerable variability in the criteria used to identify MTrPs [29] and the reliability of trigger point palpation has not been reported in the lower extremity and foot, it is imperative that researchers outline detailed diagnostic criteria used to identify MTrPs [29]. This would ensure that the methods used to diagnose MTrPs is transparent and can be reproduced.

This systematic review has a number of implications for further research. First, to reduce bias it is essential that when evaluating the effectiveness of dry needling and/or injections of MTrPs associated with plantar heel pain that rigorous randomised controlled trial (RCT) methodology be used. In addition, future RCTs should be designed based on criteria that are recognised for the quality assessment of randomised controlled trials [30]. Second, it is necessary that outcome measures used are reliable and valid and include both foot specific and generic measures [31]. Finally, it is highly recommended that the Standards for Reporting Interventions in Controlled Trials of Acupuncture (STRICTA) be used to ensure transparency. This should also include detailed information about the criteria used to identify the presence of a MTrP as there is substantial variability in the criteria used. This will ensure that such trials include sufficient information for the methodology to be critiqued and allow comparisons to be made with similar investigations.

This systematic review also needs to be viewed in light of some limitations. Two of the included trials [25,26] combined trigger point dry needling with acupuncture. While the two techniques have a number of similarities they are vastly different conceptually. Furthermore, an assessment of the effectiveness of trigger point dry needling and/or injections might be problematic when it is combined with acupuncture as it makes it difficult to isolate the effectiveness of either technique. Hence, the results can only be generalised to people with plantar heel pain where both interventions are implemented.

## Conclusions

This systematic review found limited evidence for the effectiveness of dry needling and/or injections of MTrPs associated with plantar heel pain. However, the quality of the included trials was poor and serious threats to internal validity were evident. In addition, the reporting of the methodology in these trials was inadequate, which limits comparisons with other investigations. As such it would be impossible to replicate these studies. Future trials in this area need to be parallel-group randomised controlled trials that contain adequate measures to reduce bias. Finally, it is strongly recommended that trials investigating the effectiveness of trigger point dry needling and/or injections provide detailed reporting consistent with the Standards for Reporting Interventions in Controlled Trials of Acupuncture (STRICTA).

## Competing interests

KBL is a Deputy Editor and SEM is an Associate Editor of the Journal of Foot and Ankle Research. It is journal policy that editors are removed from the peer review and editorial decision-making processes for manuscripts they have co-authored.

## Authors' contributions

The protocol for the review was written by MC. Data extraction was performed by MC and SEM. Quality analysis was performed by MC. MC, KBL and SEM drafted the review and agreed on the final manuscript. All authors read and approved the final manuscript

## Supplementary Material

Additional file 1 **Data extraction form**. Additional file 1 contains a copy of the form used to extract data from the studies included in this systematic review. Click here for file
