# Supplementary material for: Effectiveness of dry needling and injections of myofascial trigger points associated with plantar heel pain: a systematic review
Source: J Foot Ankle Res. 2010 Sep 1;3:18. doi: 10.1186/1757-1146-3-18 (PMC2942821; doi:10.1186/1757-1146-3-18)
Supplement: Additional file 1 — Data extraction form. Additional file 1 contains a copy of the form used to extract data from the studies included in this systematic review. [file 1757-1146-3-18-S1.DOC]

**Effectiveness of dry needling and injections of myofascial trigger points associated with plantar heel pain: a systematic review**

Matthew Cotchett, Karl B Landorf, Shannon E Munteanu

**Additional Data File 1. Data extraction form**

**Date**: ………………………………………………..**Assessor**: …………………………………..………**Trial title**: ……………………………………………**Journal**: ……………………………………………..

| **Setting** | **Participants** | **Blinding** | **Randomisation** | **Intervention and control** | **Outcomes** |
| --- | --- | --- | --- | --- | --- |
| Participants | Age | Single blind?  Double blind?  Researcher blind?  Subject blind?  Assessor blind? | Study described as randomised? | Dry needling and/or injection details including:   - Muscles treated - Brand of needle; - Needle gauge; - Depth of needle insertion; - Number of insertions per muscle; - Manual or electrical needle stimulation; - Response elicited. | Were the outcomes reported? |
| Inclusion criteria | Gender |  | How was randomisation sequence generated? | Treatment regimen including duration and frequency of treatment | Were the outcome measures clearly defined? |
| Exclusion criteria | Heel pain duration |  | Was there allocation concealment? How was this undertaken? | Use of co-interventions | What were the outcome measures? Are the outcome measures reliable and valid? |
|  | Number of patients in treatment and control group (mean/median characteristic values) |  | Was randomisation appropriate? | Background of the researchers | What was the time to follow up/ was the time to follow up the same for each participant? |
|  | Were treatment groups comparable at baseline? |  |  |  | Adverse affects |
|  | Baseline pain assessment? |  |  |  |  |
